# Malvern Hospital

**Published:** 1911-11-25

**Authors:** William Hanman

**Affiliations:** 19 Temple Street, Birmingham


*To the Editor of* The Hospital.

### MALVERN HOSPITAL.

*To the Editor of* The Hospital.

Sir,—The article in your issue of the 21st ult., which has only just now been brought to my notice, must have been written without an inspection of the building, probably only after a glance at the small-scale plan, otherwise the writer would have realised that the sanitary fittings are of the best description, suitably placed and equal to requirements; in fact, superior to those generally supplied to hospitals of the same size. Also that it is possible to construct an intercepting lobby, perfectly lighted and ventilated, without it being a cold and draughty passage depending on open windows each side, too often prejudicial to the health and comfort of patients and staff. The fact is precedent is too much worshipped by some who have to do with hospitals, and, without taking trouble to examine into and judge things on their merits, they unreasonably condemn originality even when it improves on what has previously been done.—Yours truly,

William Henman, F.R.I.B.A.

19 Temple Street, Birmingham, November 18.

[In reply to Mr. Henman's letter, it is perfectly true that my criticism of his plan was written without my having an opportunity of inspecting the building, but not as he suggests after "only a glance at the small scale plan." However superior to anything else the fittings may be, the fact remains that the access between the large ward and the w.c. and bath-room is a lobby which certainly can only be lighted by borrowed light from the ward and the bath-room, and apparently is not ventilated at all. Mr. Henman's letter does not explain in the least how this perfect ventilation is obtained, neither does he refer to my remark as to the bed-pan sink. If this, as I surmise, is in the bath-room, anything that Mr. Henman can say will not, in my judgment, defend such an objectionable arrangement. The last paragraph in Mr. Henman's letter is really so offensive and so uncalled for that I do not feel inclined to refer to it further.—The Writer of the Article.]

